# Carbamylated erythropoietin regulates immune responses and promotes long-term kidney allograft survival through activation of PI3K/AKT signaling

**DOI:** 10.1038/s41392-020-00232-5

**Published:** 2020-09-16

**Authors:** Ning Na, Daqiang Zhao, Jinhua Zhang, Jiaqing Wu, Bin Miao, Heng Li, Yingxun Luo, Zuofu Tang, Wensheng Zhang, Joseph A. Bellanti, Song Guo Zheng

**Affiliations:** 1 Department of Kidney Transplantation, The Third Affiliated Hospital of Sun Yat-sen University, Guangzhou, 510630 Guangdong China; 2 Department of Kidney Transplantation, The Fifth Affiliated Hospital of Sun Yat-sen University, Zhuhai, 519000 Guangdong China; 3 Thomas E. Starzl Transplantation Institute, University of Pittsburgh School of Medicine, Pittsburgh, 15261 PA USA; 4 Department of Plastic Surgery, University of Pittsburgh School of Medicine, Pittsburgh, 15261 PA USA; 5 Department of Pediatrics and Microbiology-Immunology, Georgetown University Medical Center, Washington, DC USA; 6 Department of Internal Medicine, The Ohio State University Wexner Medical Center, Columbus, 43210 OH USA; 7 Present Address: United States Army Institute of Surgical Research, JBSA Fort Sam Houston, Houston, 78234 TX USA

**Keywords:** Transplant immunology, Immunotherapy, Kidney diseases

## Abstract

Modulation of alloimmune responses is critical to improving transplant outcome and promoting long-term graft survival. To determine mechanisms by which a nonhematopoietic erythropoietin (EPO) derivative, carbamylated EPO (CEPO), regulates innate and adaptive immune cells and affects renal allograft survival, we utilized a rat model of fully MHC-mismatched kidney transplantation. CEPO administration markedly extended the survival time of kidney allografts compared with the transplant alone control group. This therapeutic effect was inhibited when the recipients were given LY294002, a selective inhibitor of the phosphoinositide 3-kinase (PI3K)/protein kinase B (AKT) signaling pathway or anti-EPO receptor (EPOR) antibody, in addition to CEPO. In vitro, CEPO inhibited the differentiation and function of dendritic cells and modulated their production of pro-inflammatory and anti-inflammatory cytokines, along with activating the PI3K/AKT signaling pathway and increasing EPOR mRNA and protein expression by these innate immune cells. Moreover, after CD4^+^ T cells were exposed to CEPO the Th1/Th2 ratio decreased and the regulatory T cell (Treg)/Th17 ratio increased. These effects were abolished by LY294002 or anti-EPOR antibody, suggesting that CEPO regulates immune responses and promotes kidney allograft survival by activating the PI3K/AKT signaling pathway in an EPOR-dependent manner. The immunomodulatory and specific signaling pathway effects of CEPO identified in this study suggest a potential therapeutic approach to promoting kidney transplant survival.

Kidney transplantation is the treatment of choice for most patients with end-stage renal disease (ESRD).^1^ Although advances in surgical procedures and immunosuppressive regimens have led to improved allograft survival, acute, and chronic rejection remain impediments to maintenance of graft function, resulting in long-term use of non-specific immunosuppressive agents.^2^ The goal of transplant investigators is therefore to identify novel strategies to promote long-term allograft survival and the avoidance of long-term immunosuppression.^3^

Erythropoietin (EPO), a glycoprotein hormone produced mainly by the kidney, is the primary regulator of erythropoiesis.^4^ EPO not only affects the hematopoietic system, but also has numerous extramedullary (non-erythropoietic) effects, such as antiapoptotic, antioxidant, and anti-inflammatory activity.^5–7^ Parsa et al.^8^ reported that preconditioning with EPO protected H9C2 myoblasts in vitro and cardiomyocytes in vivo against ischemic injury and improved cardiac function following myocardial infarction. Further reports have suggested that EPO might modulate the immune-mediated inflammatory response.^9^ In particular, recent reports that EPO inhibits conventional T cells, but promotes the induction of regulatory T cells (Treg) to improve kidney transplant survival, suggesting EPO exerts immunomodulatory effects.^10,11^ However, high doses of EPO required to achieve tissue protection increase the incidence of postoperative cardiovascular complications, especially the risk of thrombosis.^12^

To prevent the adverse effects of EPO, new generation EPO derivatives have been developed, including carbamylated EPO (CEPO), which only activate the protective EPO receptor (EPOR)—β common receptor 2 and do not stimulate erythropoiesis.^13–15^ Their safety and efficacy in the improvement of renal function following ischemia–reperfusion injury (IRI) has been demonstrated in preclinical studies.^16,17^ In addition, work by Coldewey et al.^18^ indicates that in experimental sepsis, EPO inhibits the inflammatory response through activating Janus kinase-2 (JAK2)/signal transducer and activator of transcription-6 (STAT6) and phosphoinositide 3-kinase (PI3K)/protein kinase B (AKT) signaling pathways. However, the function of CEPO-mediated PI3K/AKT signaling in modulation of the immune response following kidney transplantation remains largely unknown.

The present study aimed to address these questions using CEPO, which has been an activity spectrum that preferentially targets tissues outside the bone marrow, while maintaining similar tissue-protective effects and pharmacokinetic properties as EPO’s.^19^ We hypothesized that CEPO would regulate innate and adaptive immune cells and promote long-term renal allograft survival.

## Results

### CEPO inhibits the differentiation and function of DC and CD4^+^ T cells and activates the PI3K/AKT signaling pathway

**Fig. 1:**
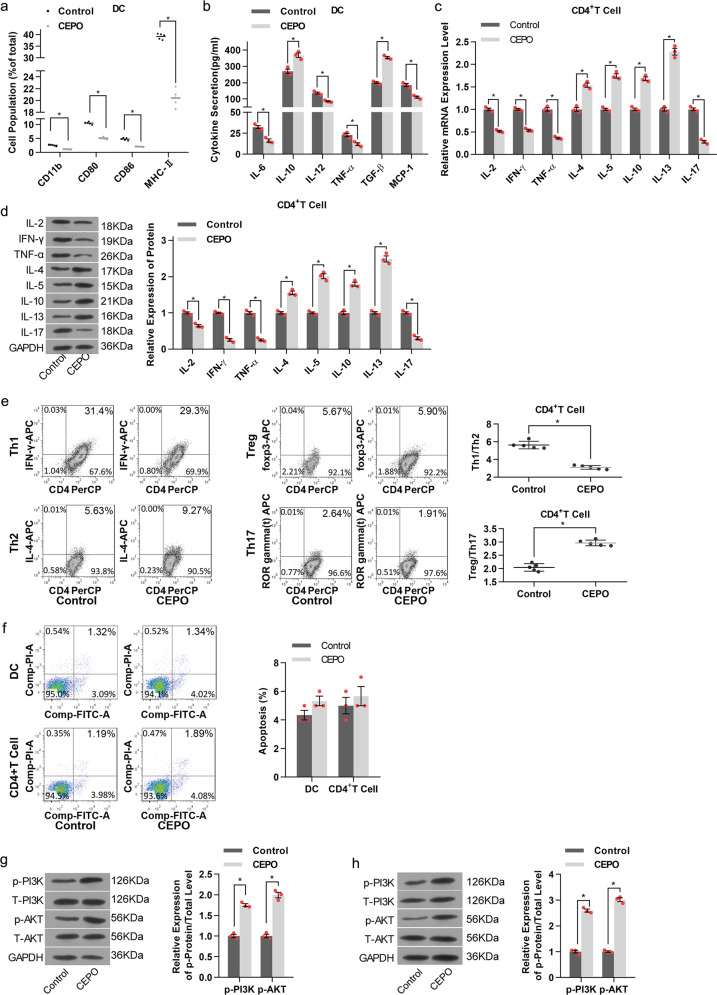
Influence of CEPO on DC and CD4^+^ T cells differentiation and the PI3K/AKT signaling pathway. **a** Incidence of CEPO on expression of CD11b, CD80, CD86, and MHC-II by DC analyzed by flow cytometry. **b** Levels of IL-6, IL-10, IL-12, TNF-α, TGF-β, and MCP-1 secreted by the CEPO-treated DC. **c** qPCR quantitation of mRNA levels of IL-2, IFN-γ, TNF-α, IL-4, IL-5, IL-10, IL-13, and IL-17 in CEPO-treated CD4^+^ T cells. **d** Western blot analysis of production of IL-2, IFN-γ, TNF-α, Th2, IL-4, IL-5, IL-10, IL-13, and IL-17 by CD4^+^ T cells. GAPDH was used to normalize each protein expression. **e** Flow analysis of Th1, Th2, Treg, Th17 cells and the ratio of Th1/Th2, Treg/Th17 cells. **f** Cell apoptosis was detected by flow cytometry with Annexin V/PI staining. **g** Western blot analysis of protein levels of p-PI3K, T-PI3K, p-AKT, and T-AKT in DC after CEPO treatment. **h** Western blot analysis of protein levels of p-PI3K, T-PI3K, p-AKT, and T-AKT in CD4^+^ T cellsafter CEPO treatment. Images shown are representative of at least three independent experiments, data are expressed as mean ± SEM (*p < 0.05, *p* values were calculated by Student’s *t*-test)

To investigate the influence of CEPO on the differentiation of conventional CD11c^+^ DC, we examined levels of CD11b, co-stimulatory molecules (CD80, CD86) and MHC-II expression by flow analysis after their exposure to CEPO for 24 h. These molecular expression levels as well as levels of the pro-inflammatory cytokines IL-6, IL-12, TNF-α, and MCP-1 were decreased significantly in the CEPO-treated group compared to the control group (Fig. 1a), whereas levels of the anti-inflammatory factors IL-10 and TGF-β increased (Fig. 1b). We also determined the influence of CEPO on the differentiation of CD4^+^ T cells by assessing Th1 (IL-2, IFN-γ, and TNF-α), Th2 (IL-4, IL-5, IL-10, and IL-13), and Th17 (IL-17) cytokine production. We found that both mRNA and protein levels of Th1 and Th17 cytokines declined markedly, while Th2 cytokines increased following exposure to CEPO (Fig. 1c, d). Furthermore, Th1 and Th17 decreased, while, Treg and Th2 were upregulated in the CEPO-treated group (Supplementary Fig. S1a). Also, the ratio of Th1/Th2 decreased and the Treg/Th17 ratio increased significantly (Fig. 1e). CEPO showed no obvious influence in cell apoptosis in both DC and CD4^+^ cells (Fig. 1f). In addition, CD4^+^ T cell cytokines, and the levels and ratios of Th1/Th2 and Treg/Th17 showed no difference in groups with or without co-culture of DCs in CEPO-treated CD4^+^ T cells (Supplementary Figs. S1e, S2a–e). Nonetheless, we found upregulated Th2 and Treg, and decreased Th1 and Th17 levels when CD4^+^ T cells were co-cultured with the DC exposed to CEPO. Correspondingly, the Th1/Th2 ratio decreased and the Treg/Th17 ratio increased significantly (Supplementary Figs. S1f, S2f, S3a–c). We then examined the expression of key proteins related to multiple signaling pathways (PI3K, AKT, NF-κB, JAK, and STAT) in DC or CD4^+^ T cells exposed to CEPO. The results showed that phosphorylated (p-) protein levels of PI3K and p-AKT were significantly higher in both CEPO-treated DC and CD4^+^ T cells than in control cells (Fig. 1g). Expression of both phosphorylated and total NF-κB, JAK, and STAT protein were unaffected by exposure of those cells to CEPO (data not shown). Together, these findings indicated that CEPO inhibited the maturation of DC, suppressed the differentiation of CD4^+^ T cells into Th1 and Th17 cells, but enhanced the prevalence of Treg and activated the PI3K/AKT signaling pathway in DC and CD4^+^ T cells.

### Pharmacological (LY294002) and siRNA-mediated inhibition of PI3K/AKT signaling blocks the inhibitory effect of CEPO on immune cells

**Fig. 2:**
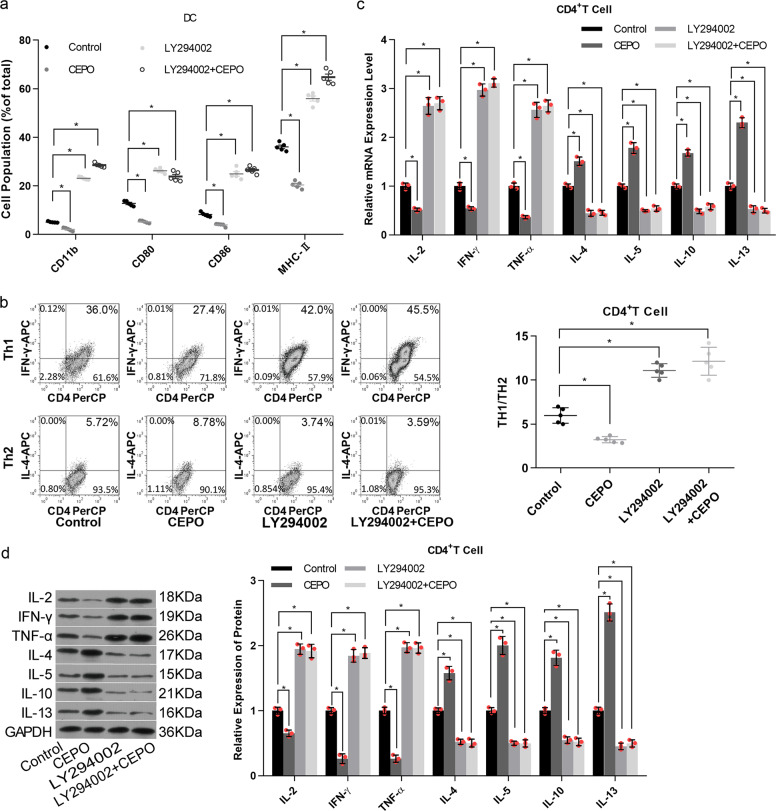
Influence of the PI3K/AKT signaling pathway on expression of pro-inflammatory cytokines determined by adding LY294002 to DC and CD4^+^ T cells. **a** Flow cytometric analysis of cell surface CD11b, CD80, CD86 and MHC-II expression by DC after addition of LY294002 ± CEPO. **b** The influence of LY294002 ± CEPO on the Th1, Th2, and Th1/Th2 ratio was determined by flow cytometry. **c** qPCR analysis of mRNA expression levels of IL-2, IFN-γ, TNF-α, IL-4, IL-5, IL-10, and IL-13 in CD4^+^T cells treated with LY294002, CEPO, or LY294002+CEPO, as indicated. **d** Protein expression of IL-2, IFN-γ, TNF-α, IL-4, IL-5, IL-10, and IL-13 in CD4^+^T cells treated with LY294002, CEPO, or c LY294002+CEPO was examined by western blot assay. GAPDH was used to normalize each protein expression. Images shown are representative of at least three independent experiments, data are expressed as mean ± SEM (*p < 0.05, *p* values were calculated by Student’s *t*-test)

We next examined the roles of PI3K/AKT signaling in the inhibitory effect of CEPO on DC and CD4^+^ T cells. LY294002, a selective PI3K inhibitor that acts on the ATP-binding site of the PI3K enzyme, was used to suppress activation of the PI3K/AKT signaling pathway. Levels of CD11b, CD80, CD86, and MHC-II expression by DC were all increased in the LY294002-treated group when compared with the control group, with or without CEPO (Fig. 2a). Moreover, the relative incidence of Th1 cells and production of the cytokines IL-2, IFN-γ, and TNF-α were reversed by LY294002, whereas Th2 cells and their associated cytokines IL-4, IL-5, IL-10, and IL-13 were increased (Fig. 2b–d, Supplementary Fig. S1b).

**Fig. 3:**
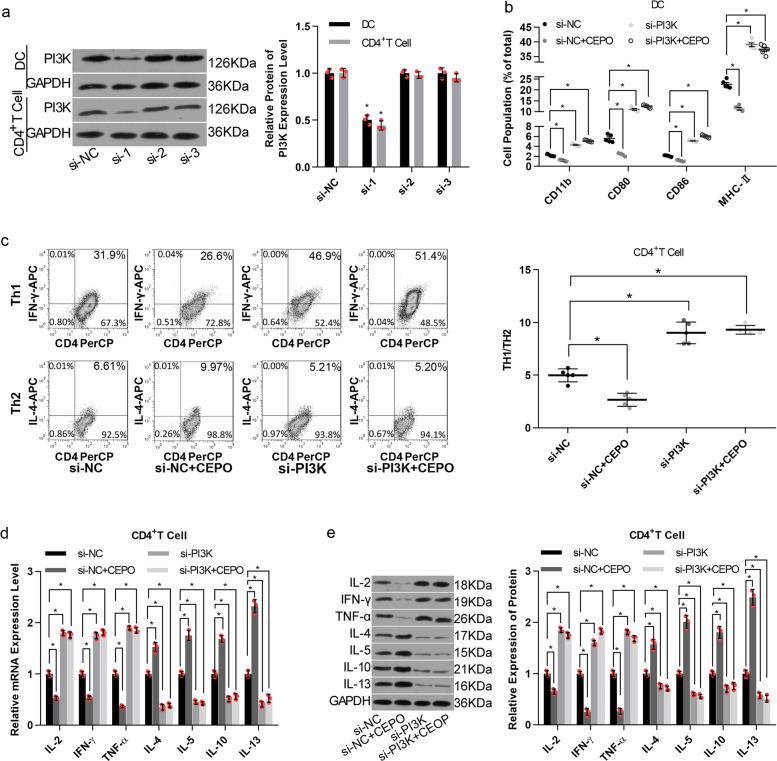
Influence of siRNA-PI3K ± CEPO on the phenotype and function of immune cells. **a** Western blot analysis of the efficiency of PI3K inhibition in DC and CD4^+^T cells after 48 h of cell transfection. **b** Flow cytometric analysis of the percentages of cell population with positive expression for CD11b, CD80, CD86, or MHC-II in DCs treated with siRNA control, siRNA control + CEPO, siRNA-PI3K, or siRNA-PI3K+CEPO, as indicated. **c** The influence of siRNA-PI3K and CEPO on the Th1, Th2, and Th1/Th2 ratio were determined by flow cytometry. **d**, **e** qPCR and western blot analysis of mRNA and protein level expression of IL-2, IFN-γ, TNF-α, IL-4, IL-5, IL-10, and IL-13 in CD4^+^ T cells treated with siRNA control, siRNA control + CEPO, siRNA-PI3K, or siRNA-PI3K+CEPO. GAPDH was used to normalize each protein expression. Images shown are representative of at least three independent experiments, data are expressed as mean ± SEM (*p < 0.05, *p* values were calculated by Student’s *t*-test)

To further explore the influence of CEPO/PI3K on immune responsiveness, immune cells were transfected with siRNA-1, siRNA-2, or siRNA-3, all targeting the rat PI3K gene. siRNA-1 showed the best knockdown efficiency in both DC and CD4^+^T cells (Fig. 3a). siRNA-PI3K transfection abolished CEPO-mediated reduction in the expressions of CD11b, CD80, CD86 or MHC-II in DC (Fig. 3b), as well neutralized CEPO roles in ratio of Th1 to Th2 levels (Fig. 3c, Supplementary Fig. S1c), and their related cytokine production (Fig. 3d, e). Taken together, these results showed that both endogenous and exogenous inhibition of PI3K/AKT signaling abolished the repressive influence of CEPO on immune cell responses.

### CEPO increases EPOR expression by DC and CD4^+^ T cells with its inhibitory effect being dependent on its interaction with EPOR

**Fig. 4:**
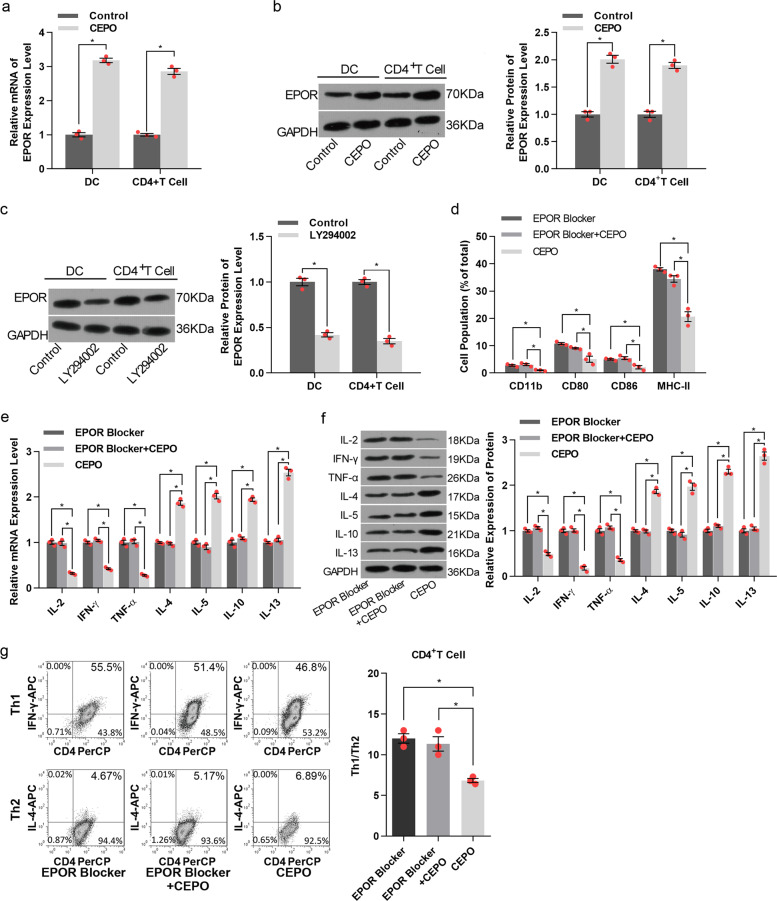
Analysis of EPO receptor (R) levels and the influence of EPOR blocker on DC and T cells. **a** Effect of CEOP on EPOR mRNA levels in DC and CD4^+^ T cells was examined by qPCR analysis. **b** Effect of CEOP on EPOR protein levels in DC and CD4^+^ T cells was detected by western blotting. **c** Western Blot analysis of EPOR protein levels after treatment of cells with the selective PI3K/AKT signaling pathway inhibitor LY294002. **d** DC cells were treated with CEOP, EPOR blocker, or CEOP+EPOR blocker, as indicated. The expression of CD11b, CD80, CD86, and MHC-II in DC were evaluated by flow cytometric analysis. **e** qPCR analysis of the mRNA levels of IL-2, IFN-γ, TNF-α, IL-4, IL-5, IL-10 and IL-13 in CD4^+^ T cells treated with the CEOP, EPOR blocker, or CEOP+EPOR blocker, as indicated. **f** Western Blot analysis of IL-2, IFN-γ, TNF-α, IL-4, IL-5, IL-10, and IL-13 protein expression in CD4^+^ T cells treated with EPOR blocker, EPOR blocker + CEPO, or CEOP alone. **g** Influence of EPOR blocker, CEPO alone, and EPOR blocker + CEPO on the Th1, Th2, and Th1/Th2 ratio was determined by flow cytometry. Images shown are representative of at least three independent experiments, data are expressed as mean ± SEM (*p < 0.05, *p* values were calculated by Student’s *t*-test)

As shown in Fig. 4a, b, expression of both EPOR mRNA and protein levels was increased markedly and significantly when DC or CD4^+^ T cells were exposed to CEPO. Additionally, expression of EPOR by these cells was decreased by exposure to the PI3K/AKT signaling pathway inhibitor LY294002 (Fig. 4c), suggesting the PI3K/AKT signaling regulated CEPO-activated EPOR expression. To explore the influence of EPOR on CEPO inhibition of immune cells, an EPOR blocking agent was used. The results showed that the levels of CD11b, CD80, CD86, and MHC-II were significantly decreased in DC (Fig. 4d). mRNA and protein expression of cytokines (Fig. 4e, f) and cells and ratios of Th1/Th2 (Fig. 4g, Supplementary Fig. S1d) in CD4^+^ T cells were all significantly altered in CEPO+EPOR blocker group as compared with the CEPO alone group, And no significant difference was shown between the CEPO+EPOR blocker and EPOR blocker alone groups. These results indicated that EPOR was essential for CEPO function in immunomodulation and the endogenous EPO is insufficient for this immunomodulation mechanism.

### CEPO prolongs renal allograft survival by activating PI3K/AKT signaling in vivo

**Fig. 5:**
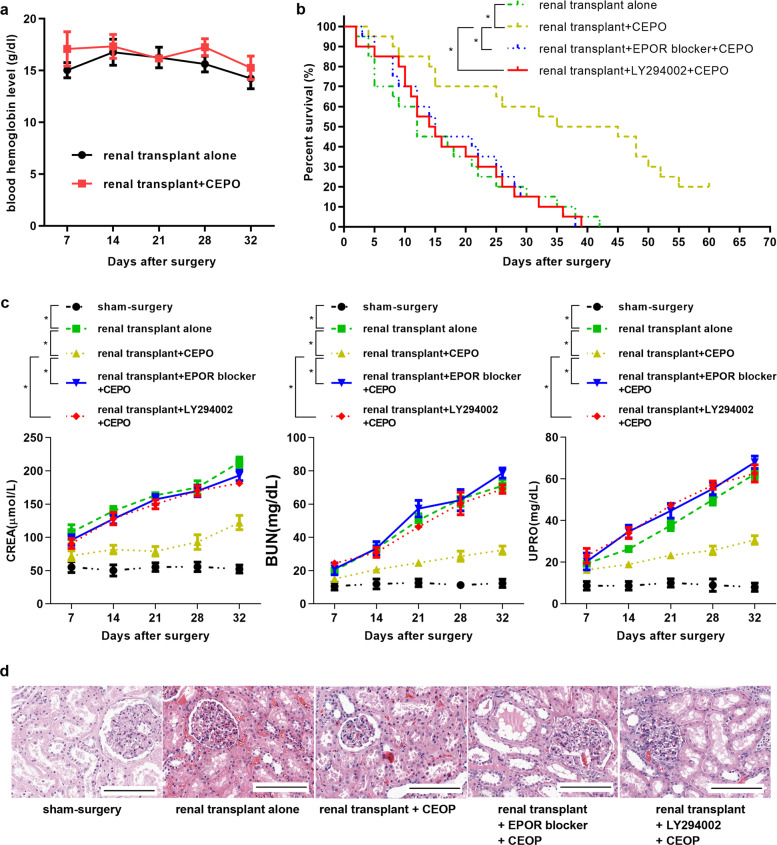
Influence of CEPO and the EPOR blocker or the PI3K/AKT inhibitor LY294002 and CEPO on renal allograft function and survival. **a** Blood hemoglobin levels of rat in renal transplant alone group and renal transplant + CEPO group were tested by ELISA (five rats per group per time point). **b** The survival curves of allografts with different treatments (*n* = 20 recipients/group) was compared: renal transplant alone, renal transplant + CEPO, renal transplant + EPOR blocker + CEPO, and renal transplant + LY292004 + CEPO. **c** The expression levels of CREA, BUN and UPRO of recipients in different groups (five rats per group per time point) are shown. **d** H & E staining showing the kidney morphology of rat from different groups (21 days after surgery): sham-surgery, renal transplant alone, renal transplant + CEPO, renal transplant + EPOR blocker + CEPO, and renal transplant + LY292004 + CEPO. Scale bar, 100 µm. The representative images of each group from five samples were shown. Data are expressed as mean ± SEM (*p < 0.05, statistical significance for continues variables was estimated by Student’s *t*-test, survival curves were compared by Log-rank test)

We also evaluated the influence of CEPO on blood hemoglobin levels in vivo and found that there was no significant change in hemoglobin levels in renal transplant + CEPO rats up to 32 d following surgery compared with animals given renal transplants alone (Fig. 5a). Next, to investigate the influence of CEPO on renal allograft survival and potential underlying mechanisms, kidney graft recipients were divided into four groups: renal transplant alone, renal transplant + CEPO, renal transplant + EPOR blocker + CEPO, and renal transplant + LY292004 + CEPO. CEPO significantly prolonged allograft median survival time (MST: 40 days, versus 12 days with renal transplant alone; *p* < 0.01), with 20% of recipients surviving >60 days (Fig. 5b) and with significantly reduced levels of CREA, BUN, and UPRO (Fig. 5c). However, levels of CREA, BUN, and UPRO, and prolonged graft survival were reversed in transplant recipients given the EPOR blocker (MST: 15 days; *p* < 0.01) or LY294002 (MST: 14.5; *p* < 0.01) in addition to CEPO. Moreover, histological evidence of rejection was detected in kidney allografts of the renal transplant alone group on 21 days and 32 days after surgery, showing marked hypertrophy of glomeruli, disordered cell arrangement, and mononuclear infiltration, and these were markedly alleviated in CEPO-treated recipients; however, the alleviation induced by CEPO was counteracted when the recipients were given EPOR blocker or LY294002 (Fig. 5d, Supplementary Fig. S6a). These results suggest that CEPO improves renal allograft function by activating the PI3K/AKT signaling pathway and is EPOR dependent.

**Fig. 6:**
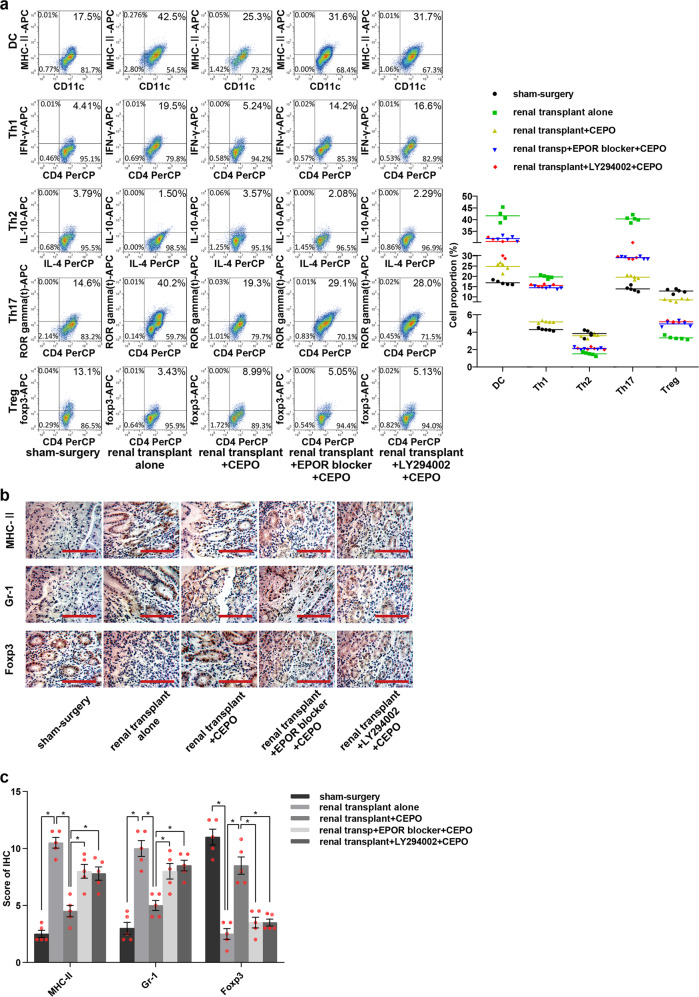
Influence of CEPO/PI3K/AKT on DC, Th1, Th2, Th17, and Treg and expression of MHC-II, Gr-1 and Foxp3. **a** Flow cytometric analysis of percentages of DC, Th1, Th2, Th17, and Treg in peripheral blood of rats from different groups (21 days after surgery): sham surgery, renal transplant alone, renal transplant + CEPO, renal transplant + EPOR blocker + CEPO, and renal transplant + LY292004 + CEPO. **b** Immunohistochemistry analysis of the protein expression of MHC-II, Gr-1, and Foxp3 in the kidney tissues from different groups (21 days after surgery): sham-surgery, renal transplant alone, renal transplant + CEPO, renal transplant + EPOR blocker + CEPO, and renal transplant + LY292004 + CEPO. Scale bar, 100 µm. **c** Immunohistochemistry staining scores for MHC-II, Gr-1, and Foxp3 in different groups. The representative images of each group from five samples were shown. Data are expressed as mean ± SEM (*p < 0.05, *p* values were calculated by Student’s *t*-test)

**Fig. 7:**
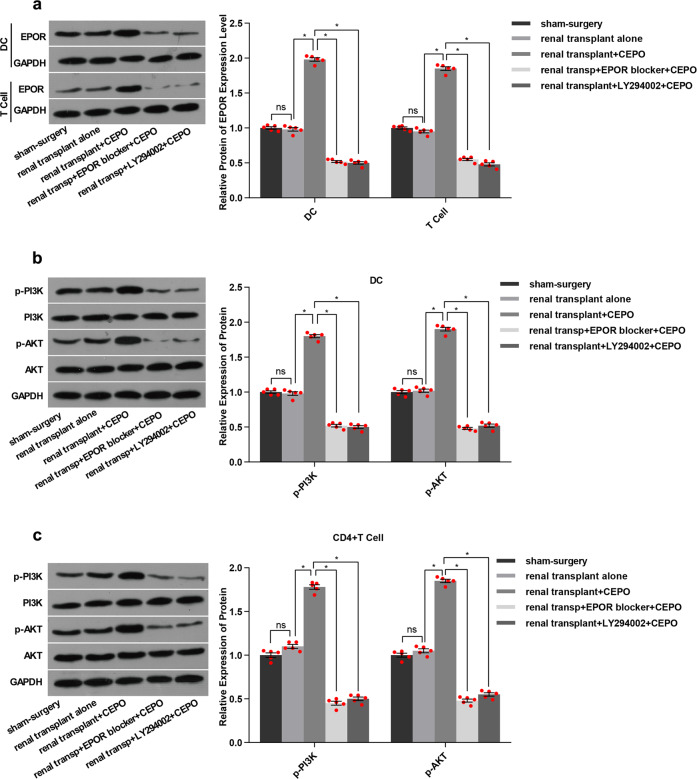
Influence of CEPO, EPOR blocker, and PI3K/AKT inhibitor on Protein levels of EPOR, PI3K, and AKT. **a** Protein expression of EPOR in DC and CD4^+^ T cells from renal transplant recipient. **b** Protein expression of PI3K and AKT in DC from renal transplant recipients. **c** Protein expression of PI3K and AKT in CD4^+^ T cells from renal transplant recipients. GAPDH was used to normalize each protein expression. The representative images of each group from 5 samples were shown. Data are expressed as mean ± SEM (*p < 0.05, *p* values were calculated by Student’s *t*-test)

The numbers of mature DC, Th1, and Th17 cells in the peripheral blood of renal transplant alone group were all increased significantly after 21 and 32 days of surgery, whereas Th2 and Treg numbers were decreased (data from 32 days post-transplant are shown in supplementary figures) when compared with the sham-surgery group. Surprisingly, the numbers of cells showed no significant differences among groups 7 days post-transplant (data are shown in supplementary figures). However, the numbers of DC, Th1, and Th17 were reduced and Th2 and Treg numbers were increased 21, 32 days post-transplant when graft recipients were given CEPO. This effect was reversed when the animals were given LY294002 or EPOR blocker in addition to CEPO (Fig. 6a, Supplementary Fig. S6c). Immunohistochemistry was performed to evaluate the expression of DC maturation markers (MHC-II), neutrophil granulocyte (Gr-1), and Treg (Foxp3), respectively in the transplanted tissue from each group. The results (Fig. 6b, c, Supplementary Fig. S6b) showed that levels of MHC-II and Gr-1 were elevated and Foxp3 decreased in the transplant alone control group and that CEPO reversed these effects. However, the influence of CEPO in inhibiting the expression of MHC-II and Gr-1, as well as promoting Foxp3 expression were impaired when the rats were administrated LY294002 or EPOR blocker. In addition, protein levels of EPOR (Fig. 7a, Supplementary Fig. S7a), and p-PI3K and p-AKT (Fig. 7b, c, Supplementary Fig. S7b, c) were upregulated markedly in DC and CD4^+^ T cells of transplanted rats given CEPO, but these changes were reversed when the animals were given LY294002 or EPOR blocker. Taken together, these data indicated that CEPO promoted long-term renal allograft survival via EPOR and activation of the PI3K/AKT signaling pathway.

## Discussion

Allograft rejection involves a complex sequence and array of events that include both innate and adaptive cellular immune responses. The present study reports the impact of CEPO, a synthetic EPO derivative, on innate and adaptive immune cells and kidney allograft survival and underlying mechanisms. While it has been reported that CEPO generated by reaction of EPO with potassium cyanate can trigger EPO-mediated tissue-protective pathways without cross-talk with the hematopoietic system,^19^ these new results now show that the CEPO can promote allograft survival by activating the PI3K/AKT signal pathway.

Immune-mediated rejection is the major barrier to successful transplantation and improved, long-term allograft survival. DC and CD4^+^ T cells are important instigators of rejection and graft destruction, but also conversely, exert immune regulatory effects that can promote immune tolerance.^20,21^ The present study demonstrates that CEPO can restrain DC and proinflammatory CD4^+^ T cell development and inhibit their release of key pro-inflammatory mediators. CEPO not only was shown to have direct effects on DC and CD4^+^ T cell but also indirectly regulated T cell differentiation via restraining maturation of DC. DC constitute a heterogeneous cell population, that range from phenotypically and functionally immature to mature antigen-presenting cells.^22^ Their ability to integrate and regulate innate and adaptive immunity is dependent on proinflammatory and anti-inflammatory environmental factors. Many reports have shown that immature DC can induce T cell unresponsiveness in vitro and in vivo, including in transplant recipients.^23–25^ We have reported previously that immature DC can increase numbers of Treg and stimulate Th2 cytokines via indoleamine 2, 3-dioxygenase production and prolong kidney allograft survival.^26^ CD4^+^ T cells play a vital role in transplant rejection and it has become clear that they have heterogeneous properties dependent on diverse subsets.^21^ Regulation of Th1/Th2 responses also plays an important role in autoimmune diseases.^27–29^ Treg can promote tolerance,^30^ whereas Th17 cells mediate inflammatory responses.^31^ Our study demonstrates for the first time, that CEPO can promote allograft survival through decreased the levels of CD11b, CD80, CD86, and MHC-II expression by DC, promotion of the expression of anti-inflammatory cytokines (IL-4, IL-5, IL-10, and IL-13) and inhibition of pro-inflammatory cytokine (IL-2, IFN-γ, IL-17, and TNF-α) expression by CD4^+^ T cells. Meanwhile, no significant influence by CEPO on hematopoiesis was found when monitoring blood hemoglobin levels of graft recipients following kidney transplantation. However, previous studies^32^ demonstrated that EPO exerts an opposite role from CEPO in regulating the immunostimulatory properties of immature DC and actually increased murine splenic DC population in vivo. We consider that the differences in molecular structures of CEPO and EPO may have contributed to these contradictory effects. EPO has been shown to intensify the sensitivity of DCs to stimulation by the TLR-4 ligand.^33^ Additionally, EPO is capable of increasing hemoglobin levels, which can up-regulate CD86 expression of DC.^34^ It has been reported that EPO inducer, which acts as an EPO/EPOR enhancer, can enhance mitochondrial biogenesis and hemoglobin production in non-haematopoietic cells, such as bone marrow cells, regulating intracellular energetics by facilitating tissue oxygen or enhancing cellular oxygenation.^35^ These findings highlight the possible mechanisms by which the haematopoietic function of EPO may play a role in its action on immune function.

Our study shows that EPOR was essential for CEPO to exert its anti-inflammatory effect. CEPO did not play any role in DC differentiation or affect the levels of cytokines related to inflammatory responses when the EPOR blocker was given to transplant recipients. It has been reported that the reaction between EPO and EPOR homodimers activates mitogen-activated kinases and NF-κB in erythroid cells and decreases production of TNF-α, a pro-inflammatory factor.^36,37^ Under hypoxic/ischemic conditions, EPO strongly stimulates the expression of EPOR and the signal from EPOR in endothelial cells is increased. It has been reported that endothelial cells expressing EPOR are the first cells to serve as targets of the extra-hematopoietic activities of EPO during angiogenesis.^38^ The present study also shows that CEPO upregulates the in vitro and in vivo immune expression of EPOR that is then involved in immune modulation/regulation induced by the CEPO.

We observed that inhibition of PI3K by siRNA or LY294002 abolished the therapeutic effect of CEPO. Previous studies have demonstrated that PI3K/AKT is the downstream signaling pathway of EPO. EPO has been reported to attenuate renal, intestinal, and myocardial IRI by suppressing inflammation, which was associated with activation of PI3K/AKT signaling.^39–41^ Similarly, in the current study, we found that CEPO activated PI3K/AKT signaling, but showed no significant influence in the expression of phosphorylated and total JAK2, STAT6, and NF-κB. However, EPO has been shown to activate multiple EPOR signaling pathways (AKT, MAPK, and NF-κB) that aid the survival, maturation, and proliferation of DC.^32,42^ Our results suggest that CEPO is distinct from EPO in that it regulates DC function by specifically targeting PI3K/AKT activation, while not affecting other classical EPO-mediated signaling mechanisms, thus inhibiting alloimmune responses.

In conclusion, the present study demonstrated that CEPO promoted long-term graft survival by activating PI3K/AKT and EPOR-dependent signaling, closely associated with modulation of the host proinflammatory and anti-inflammatory immune responses. These findings point to a new therapeutic approach to the promotion of long-term organ allograft survival and the development of drugs with a novel activity spectrum that specifically targets protective EPOR.

## Materials and methods

### Animals

Male Lewis and Brown Norway rats weighing 250–350 g were purchased from the Beijing Vital River Laboratory Animal Technology Co., Ltd (SPF; Beijing, China) and maintained under specific pathogen-free conditions. Animal procedures were performed in accordance with the guidelines of the Animal Care and Use Committee of Sun Yat-Sen University.

### Kidney transplantation

The transplant procedure was performed as described.^43^ Kidneys from donor Brown Norway rats were transplanted to the right of recipient Lewis rats (age 12–14 weeks) with a left nephrectomy. Sham surgeries were performed without nephrectomy. Animals were divided into five groups: sham-surgery, renal transplant alone, renal transplant + CEPO, renal transplant + EPOR blocker + CEPO, and renal transplant + LY294002 + CEPO. CEPO (Chugai Pharmaceuticals, Tokyo, Japan; 30 μg/kg), EPOR blocker (M-20, 100 μg/kg, Santa Cruz Biotechnolgy, TX), LY294002 (1 mg/kg, MedChemExpress, NJ) were given once daily via tail vein injection for successive 3 days. Blood samples were collected 7, 14, 21, 28, and 32 days after kidney transplantation for measurement of hemoglobin levels and renal function by monitoring levels of serum creatinine (CREA), blood urea nitrogen (BUN), and urinary protein (UPRO) using a Beckman AU480 Chemistry Analyzer. Five rats from each group were euthanized at each time point for biochemical and immunological assays (totally 125 rats). Twenty recipient rats in each transplanted group (total 80 rats) were used to determine renal allograft survival.

### Cell preparation and treatment

Single-cell suspensions obtained from freshly-isolated bone marrow of tibias and femurs of Lewis rats were cultured in RPMI-1640 medium containing 10% v/v FBS (Invitrogen) at a concentration of 5 × 10^6^ cells/ml in 6-well plates. Three hours later, non-adherent cells were discarded and new medium supplemented with rat recombinant granulocyte-macrophage colony-stimulating factor (GM-CSF) (10 ng/ml) and interleukin (IL)-4 (10 ng/ml) (PeproTech, NJ) was added. For generating dendritic cells (DC), fresh medium and cytokines were added to the cultures every 3 days. Loosely-adherent cell clusters were harvested on days 6–8 and DC were purified using flow sorting with anti-rat CD11c Ab (BD Biosciences). CD4^+^ T cells were isolated from Lewis rat blood and purified using CD4 MicroBeads (Miltenyi Biotec, Germany). The isolated CD4^+^ T cells were cultured in regular medium containing 89% RPMI-1640 media, 10% FBS and 1% penicillin-streptomycin. DC and CD4^+^ T cells were incubated with CEPO (150 ng/ml) for 24 h. The quantitation of Th1, Th2, Treg, and Th17 populations was measured after treatment of CD4+ T with CEPO for 5 days or without treatment. To inhibit the PI3K/AKT signaling pathway or EPOR, cells were incubated with LY294002 (20 μΜ, Calbiochem, CA) for 1 h or EPOR blocker (50 μM, Merck, CA) for 2 h, respectively. To downregulate PI3K expression, small interfering RNAs (siRNAs, OriGene, Beijing, China) targeting the rat PI3K gene were recruited and cells were analyzed 48 h post-transfection.

### Co-culture of CD4^+^ T cells and DC

The isolated CD4^+^ T and DC populations were co-cultured using a transwell culture system (BD Bioscience) using two experimental systems. In the first system, CD4^+^ T cells were seeded in the lower chamber of a 24-well transwell culture system at a density of 1 × 10^4^ cells/well with or without DCs (1 × 10^4^ cells/well) in the upper chamber. Then, CEPO (150 ng/ml) was added into the lower chamber and the cells were incubated at 37 °C for 24 h following which, CD4^+^ T cells were harvested. In the second system, DCs (1 × 10^4^ cells/well) with or without CEPO (150 ng/ml) treatment were inoculated in the upper chamber of a 24-well transwell culture system and CD4^+^ T cells were seeded in the lower chamber at a density of 1 × 10^4^ cells/well following which the chamber was incubated at 37 °C for 24 h. After that, CD4^+^ T cells were collected.

### Flow cytometric analysis

Cultured cells or cells harvested from peripheral blood were stained with fluorochrome-conjugated anti-rat CD4, CD25, CD80, CD86, CD11c, CD11b, IL-4, MHC-II, IFN-γ, and ROR-γt, Foxp3 Abs (BD Biosciences) and analyzed for expression of various cell surface or intracellular markers using a FACSCalibur platform (BD Biosciences). For intracellular cytokine (IL-4, IFN-γ) detection, cells were cultured with cell stimulation cocktail (protease inhibitor) for 16 h, then fixed and permeabilized with Fixation/Permeablization Buffer (eBioscience, MA) according to the manufacturer’s protocol. CD4^+^ T cell subsets were defined by expression of CD4 and IFN-γ for T helper (Th)1, expression of CD4 and IL-4 for Th2, expression of CD4 and ROR-γt for Th17 and expression of CD4, CD25, and Foxp3 for Treg. Cell apoptosis was tested using the Annexin V-FITC Apoptosis Detection Kit I (BD Biosciences). For the enumeration of peripheral blood DC and CD4^+^ T cell subsets, CountBright absolute count beads (ThermoFisher Scientific, MA) were mixed with the cell samples and assayed via flow cytometry. Data were analyzed using FlowJo software (Tree Star, OR).

### Western blot analysis

Total protein was obtained from tissues or cultured cells using radioimmunoprecipitation assay buffer (Sangon Biotech, China). Equal protein from each sample was separated on a 10% SDS-PAGE gel and transferred onto a polyvinylidene difluoride membrane (Bio-Rad, CA). The membranes were incubated overnight at 4 °C with the primary Abs against IL-2, IFN-γ, TNF-α, IL-4, IL-10, IL-13, p-PI3K (Abcam, CA), PI3K, AKT, p-AKT (Cell Signaling Technology, CA) and IL-5 (R&D, CA), and incubated with the corresponding secondary Abs. Immune complexes were examined by enhanced chemiluminescence detection and quantified by ImageJ software (National Institutes of Health).

### Quantitative polymerase chain reaction (qPCR) assay

Total RNA from DC and CD4^+^ T cells was extracted using an RNApure Tissue Kit (Millipore, Darmstadt, Germany) and reverse-transcribed to cDNA using the HiFiScript 1st Strand cDNA Synthesis Kit (CWBIO, Beijing, China). Real-time PCR was performed using TransStart Green qPCR SuperMix (Transgen, Beijing, China) and tested using the DA7600 real-time nucleic acid amplification fluorescence detection system (Bio-Rad, CA). Primers synthesized by the Beijing Genomics Institute are listed in Supplementary Table 1. Data are expressed using the *C*_T_ method and normalized against housekeeping gene (GAPDH) levels.

### ELISA

Levels of cytokines (IL-6, IL-10, IL-12, TNF-α, TGF-β, and monocyte chemotactic protein-1 [MCP-1]) in culture supernatants were measured using ELISA kits (Abcam, CA) following the manufacturer’s instructions.

### Histology and immunohistochemistry

Kidney sections (4 μm) were stained with hematoxylon & eosin (HE) for morphological evaluation. In addition, a routine, three-step immunohistochemical staining procedure was carried out with primary Abs against MHC-II (Abcam), Gr-1 (Bio-Rad), or Foxp3 (Abcam). Samples were evaluated in a blinded fashion at two to three different levels of sectioning according to the staining extent and intensity. The extent of staining was scored by the percentage of the positively stained area: 0 for a percentage <5%, 1 for 5–25%, 2 for 25–50%, 3 for 50–75%, and 4 for 75%. The staining intensity was scored as 0, 1, 2, and 3 for the representation of negative (no staining), mild (weak), intermediate (distinct), and intense (strong) staining, respectively. The staining intensity and extent scores were multiplied to make the final score.

### Statistical analyses

Data are expressed as means ± SEM. Differences between experimental groups were analyzed by Student’s *t*-test for two groups comparisons. Kidney allograft survival were assessed using Kaplan–Meier survival analysis, and the Log-rank (Mantel–Cox) test was used to determine the statistical significance of differences. *p* values < 0.05 were considered significant for all statistical tests. The results were analyzed using Statistical Package for Social Sciences (SPSS) version 17.0 (Chicago).

## Supplementary information

Supplementary Fig S1

Supplementary Fig S2

Supplementary Fig S3

Supplementary Fig S4

Supplementary Fig S5

Supplementary Fig S6

Supplementary Fig S7

Supplementary Information

## Data Availability

All data generated or analysed during this study are included in this published article (and its supplementary information files).
