# Supplementary material for: Carbamylated erythropoietin regulates immune responses and promotes long-term kidney allograft survival through activation of PI3K/AKT signaling
Source: Signal Transduct Target Ther. 2020 Sep 16;5:194. doi: 10.1038/s41392-020-00232-5 (PMC7493938; doi:10.1038/s41392-020-00232-5)
Supplement: Supplementary file 8 — Supplementary Information [file 41392_2020_232_MOESM8_ESM.docx]

**Supplementary Information**

(This file contains supplementary table 1 and supplementary figures S1-6.)

**Carbamylated erythropoietin regulates immune responses and promotes long-term** **kidney allograft survival through activation of PI3K/AKT signaling**

Ning Na^1^**^†^** Daqiang Zhao^1^**^†^**, Jinhua Zhang^1^**^†^**, Jiaqing Wu^2^, Bin Miao^1^, Heng Li^1^, Yingxun Luo^1^,

Zuofu Tang^1^, Wensheng Zhang^3,4*^, Joseph A. Bellanti^5^ and Song Guo Zheng^6*^

**^†^** These authors contributed equally to this work

1. Department of Kidney Transplantation, The Third Affiliated Hospital of Sun Yat-sen University, Guangzhou, Guangdong Province 510630, China

2. Department of Kidney Transplantation, The Fifth Affiliated Hospital of Sun Yat-sen University, Zhuhai, Guangdong Province 519000, China

3. Thomas E. Starzl Transplantation Institute, University of Pittsburgh School of Medicine, Pittsburgh, PA 15261, USA

4. Department of Plastic Surgery, University of Pittsburgh School of Medicine, Pittsburgh, PA, 15261, USA

5. Department of Pediatrics and Microbiology-Immunology, Georgetown University Medical Center, Washington, DC, USA

6. Department of Internal Medicine, The Ohio State University Wexner Medical Center, Columbus, 43210 OH, USA

**Running Title:** CEPO for kidney transplantation

^*^ Co-Corresponding Authors:

Wensheng Zhang, M.D., Ph.D.

Thomas E. Starzl Transplantation Institute

Department of Plastic Surgery

University of Pittsburgh School of Medicine

200 Lothrop Street, E1555 Biomedical Science Tower, Pittsburgh, PA 15261, USA

Present address: United States Army Institute of Surgical Research

JBSA Fort Sam Houston, TX 78234, USA

Email: georgewen0109@gmail.com

Son Guo Zheng, M.D., Ph.D.

Department of Internal Medicine, The Ohio State University Wexner Medical Center,

480 Medical Center Dr., Columbus, 43210 OH, United States

Email: SongGuo.Zheng@osumc.edu

**Table 1** Primer sequences for qPCR

| Gene Name | Forward Sequences | Reverse Sequences |
| --- | --- | --- |
| IL-2 | CTCCCCATGATGCTCACGTT | TCCAGCGTCTTCCAAGTGAA |
| IFN-γ | CGCTACACACTGCATCTTGG | TCCTTTTGCCAGTTCCTCCA |
| TNF-α | ATGGGCTCCCTCTCATCAGT | CAAGGGCTCTTGATGGCAGA |
| IL-4 | GTACCGGGAACGGTATCCAC | GTGAGTTCAGACCGCTGACA |
| IL-5 | TGAGACGATGAGGCTTCCTG | TCAGACTTCCATTGCCCACTC |
| IL-10 | TCCGGGGTGACAATAACTGC | TGGCCTTGTAGACACCTTTGT |
| IL-13 | CATGGTATGGAGCGTGGACC | TCCGAGGCCTTTTGGTTACA |
| EPOR | AGCCCAGAGAGCGAGTTTGA | CCACAGGCAGCCATCATTCT |
| GAPDH | GCGAGATCCCGCTAACATCA | CTCGTGGTTCACACCCATCA |

**SUPPLEMENTARY FIGURE LEGENDS**


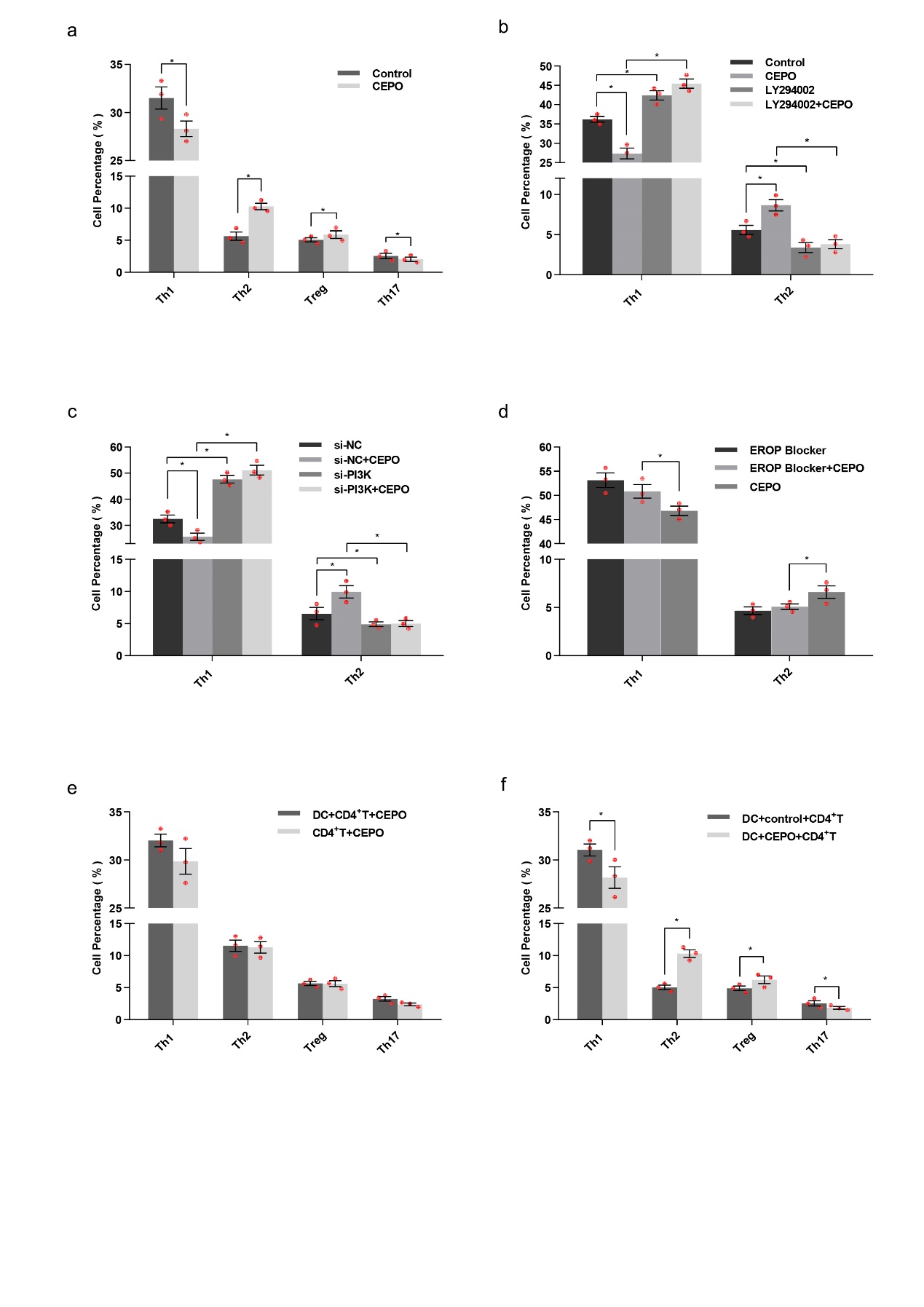


Figure S1. Cell populations of Th1, Th2, Treg, and Th17 by Flow analysis. a) Incidence of CEPO on Th1, Th2, Treg, and Th17 cells. b) The influence of LY294002 ± CEPO on the Th1 and Th2. c) The effects of siRNA-PI3K and CEPO on the Th1 and Th2. d) The influence of the EPOR blocker ± CEPO on the Th1 and Th2. e) The influence of DC on the Th1, Th2, Treg, and Th17 cells with treatment of CERO. f) The influence of DC with or without exposure to CEPO on the Th1, Th2, Treg, and Th17 cells. Images shown are representative of at least three independent experiments, data are expressed as mean ± SEM (^*^*p* < 0.05, p values were calculated by Student’s *t*-test).


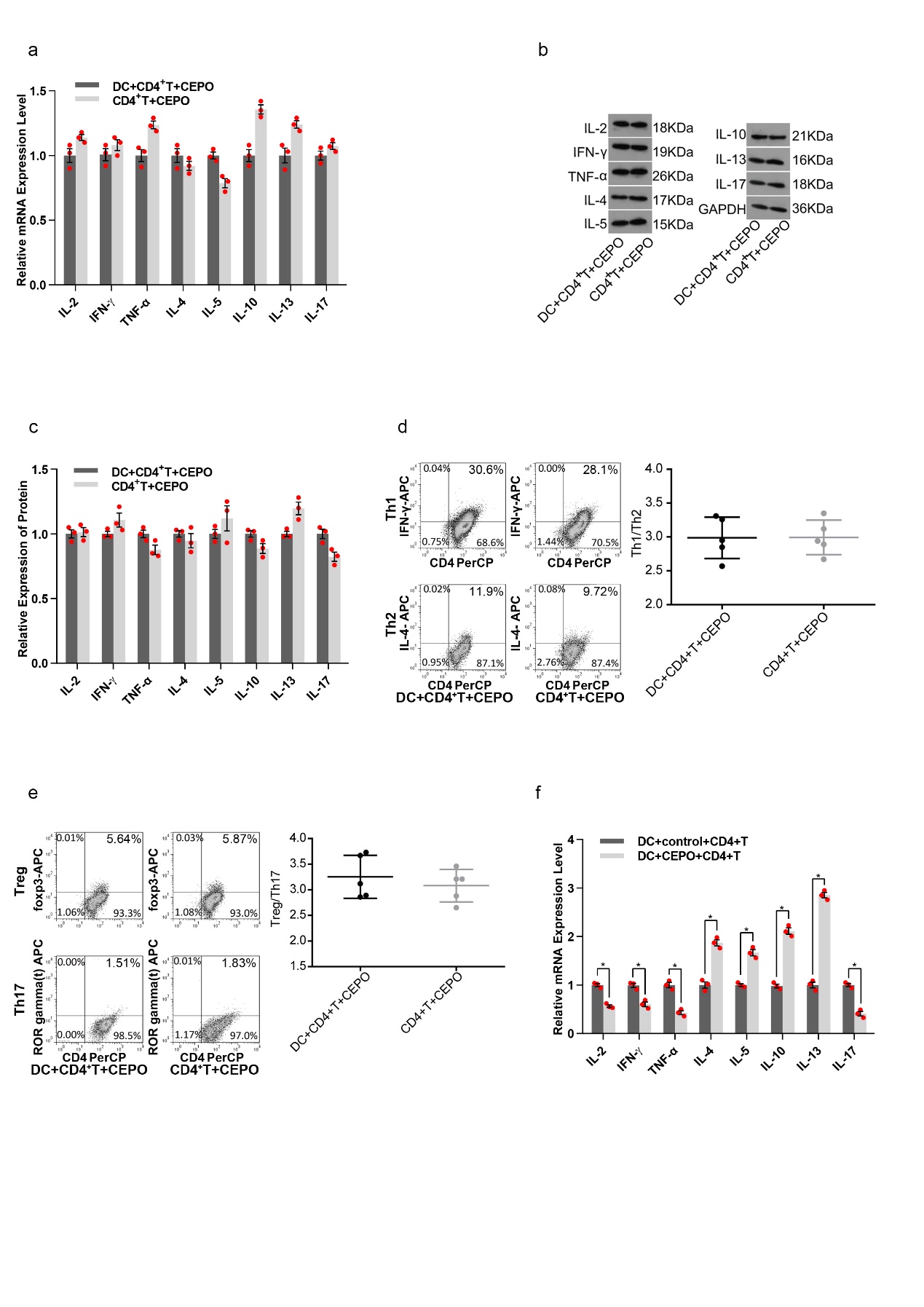


Figure S2. Influence of DC on differentiation of CD4^+^ T cells treated with CEPO. a) qPCR quantitation of mRNA levels of IL-2, IFN-γ, TNF-α, IL-4, IL-5, IL-10, IL-13, and IL-17 in CEPO-treated CD4^+^ T cells co-cultures with DC. b-c) Western blot analysis of production of IL-2, IFN-γ, TNF-α, Th2, IL-4, IL-5, IL-10, IL-13, and IL-17 by CD4^+^ T cells. GAPDH was used to normalize each protein expression. d) Flow analysis of Th1, Th2 cells and the ratio of Th1/Th2 cells. e) Flow analysis of Treg, Th17 cells and the ratio of Treg/Th17 cells. f) qPCR quantitation of mRNA levels of IL-2, IFN-γ, TNF-α, IL-4, IL-5, IL-10, IL-13, and IL-17 in CD4^+^ T cells co-cultured with CEPO-treated DC. Images shown are representative of at least three independent experiments, data are expressed as mean ± SEM (^*^*p* < 0.05, p values were calculated by Student’s *t*-test).


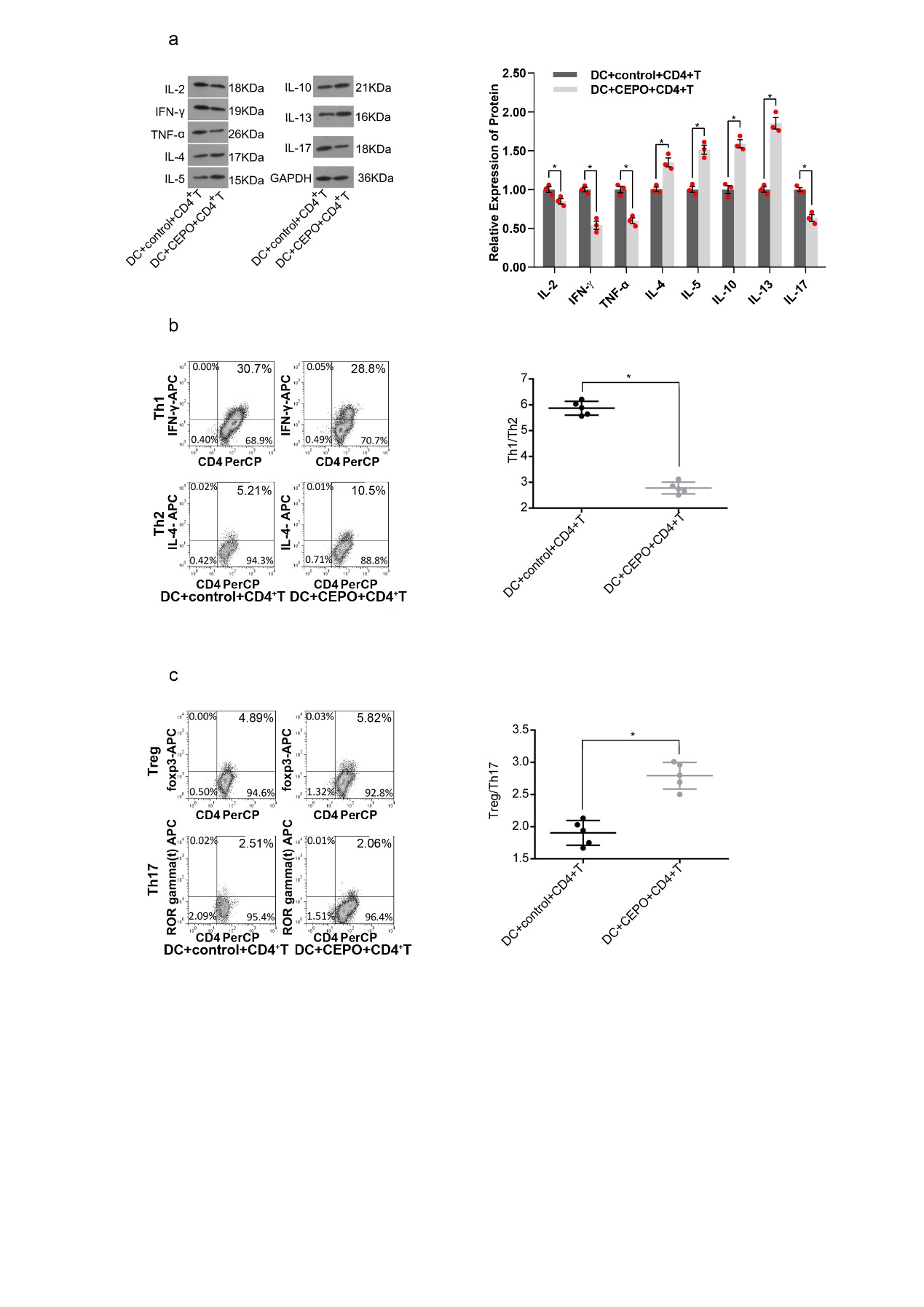


Figure S3. Influence of DC exposure to CEPO on CD4^+^ T cells differentiation. a) Western blot analysis of expression of IL-2, IFN-γ, TNF-α, Th2, IL-4, IL-5, IL-10, IL-13, and IL-17 by CD4^+^ T cells. GAPDH was used to normalize each protein expression. b) Flow analysis of Th1, Th2 cells and the ratio of Th1/Th2 cells. c) Flow analysis of Treg, Th17 cells and the ratio of Treg/Th17 cells. Images shown are representative of at least three independent experiments, data are expressed as mean ± SEM (^*^*p* < 0.05, p values were calculated by Student’s *t*-test).


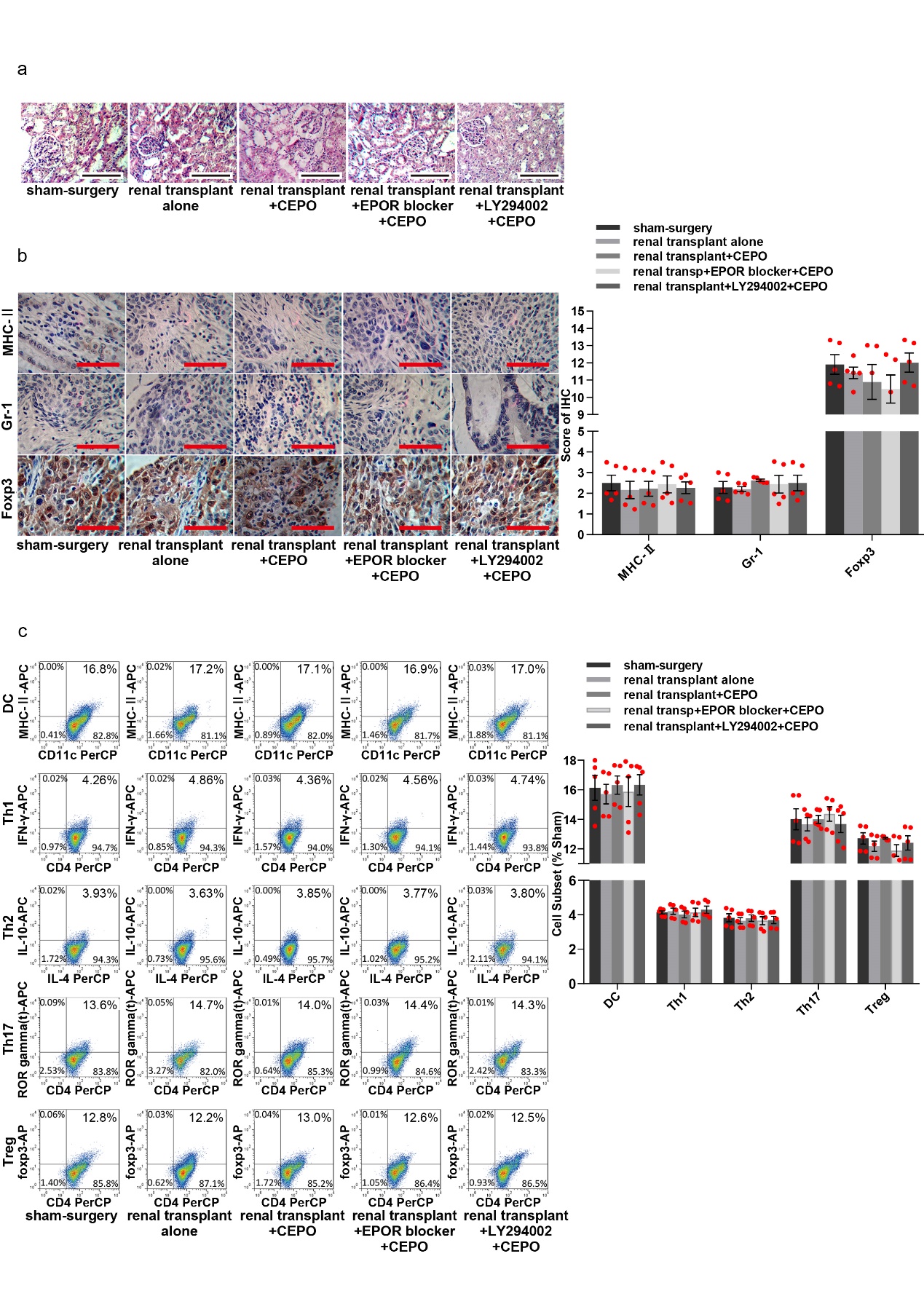


Figure S4. Influence of CEPO/PI3K/AKT on allograft function, DC, Th1, Th2, Th17 and Treg and expression of MHC-II, Gr-1 and Foxp3 7 days post-transplant. a) H & E staining showing the kidney morphology of rat from different groups (7 days after surgery): sham-surgery, renal transplant alone, renal transplant + CEPO, renal transplant + EPOR blocker + CEPO, and renal transplant + LY292004 + CEPO. b) Immunohistochemistry analysis of the protein levels of MHC-II, Gr-1 and Foxp3 in the kidney tissues from different groups (7 days after surgery): sham-surgery, renal transplant alone, renal transplant + CEPO, renal transplant + EPOR blocker + CEPO, and renal transplant + LY292004 + CEPO. Immunohistochemistry staining scores of MHC-II, Gr-1 and Foxp3 expressing levels in different groups. Scale bar, 100 µm. c) Flow cytometric analysis of numbers of DC, Th1, Th2, Th17 and Treg in rat peripheral blood from different groups (7 days after surgery): sham surgery, renal transplant alone, renal transplant + CEPO, renal transplant + EPOR blocker + CEPO, and renal transplant + LY292004 + CEPO. The representative images of each group from 5 samples are shown and data are expressed as the mean ± SEM (^*^*p* < 0.05, p values were calculated by Student’s *t*-test).


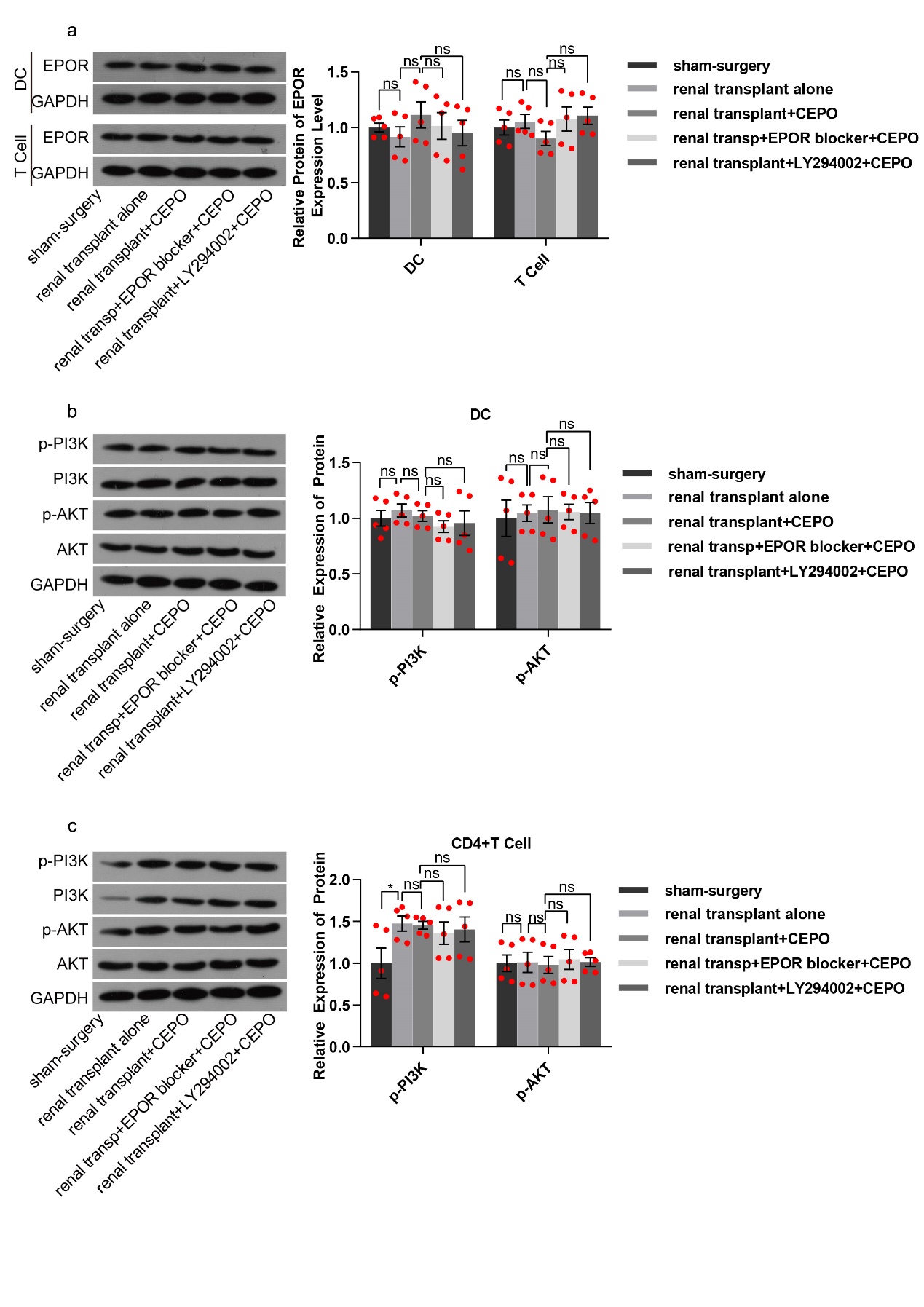


Figure S5. Influence of CEPO, EPOR blocker and PI3K/AKT inhibitor on levels of EPOR, PI3K, and AKT 7 days post-transplant. Protein expression in renal transplant recipient immune cells was assessed by western blot analysis. a) Expression of EPOR in renal transplant recipient DC and CD4^+^ T cells. b) Analysis of PI3K and AKT levels in DC of renal transplant recipients. c) Analysis of PI3K and AKT levels in CD4^+^ T cells of renal transplant recipients. GAPDH was used to normalize each protein expression. The representative images of each group from 5 samples are shown and data are expressed as the mean ± SEM (^*^*p* < 0.05, p values were calculated by Student’s *t*-test).


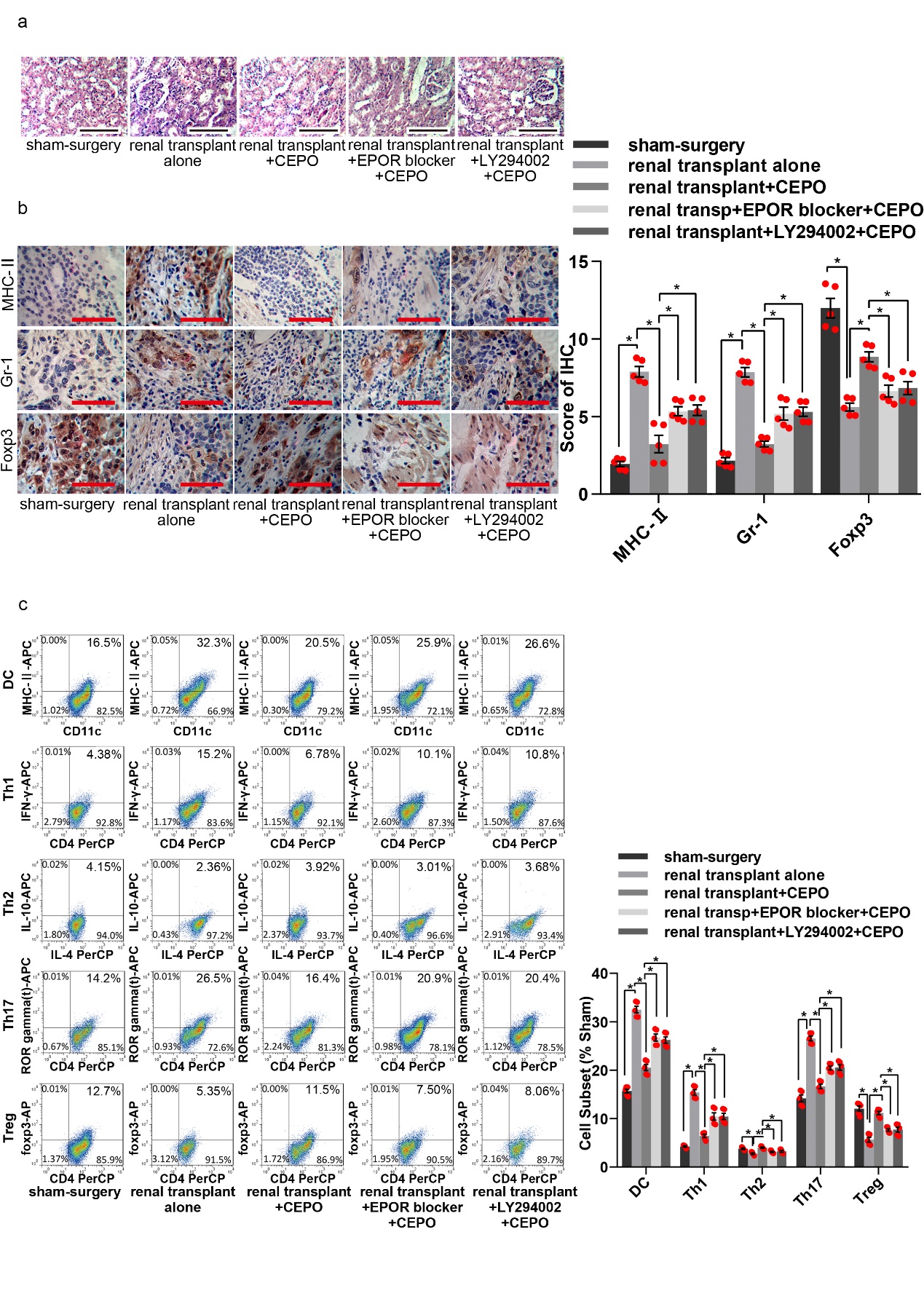


Figure S6. Influence of CEPO/PI3K/AKT on allograft function, DC, Th1, Th2, Th17, and Treg and expression of MHC-II, Gr-1 and Foxp3 32 days post-transplant. a) H & E staining showing the kidney morphology of rat from different groups (32 days after surgery): sham-surgery, renal transplant alone, renal transplant + CEPO, renal transplant + EPOR blocker + CEPO, and renal transplant + LY292004 + CEPO. b) Immunohistochemistry analysis of the protein levels of MHC-II, Gr-1 and Foxp3 in the kidney tissues from different groups (32 days after surgery): sham-surgery, renal transplant alone, renal transplant + CEPO, renal transplant + EPOR blocker + CEPO, and renal transplant + LY292004 + CEPO. Immunohistochemistry staining scores of MHC-II, Gr-1 and Foxp3 expressing levels in different groups. Scale bar, 100 µm. c) Flow cytometric analysis of numbers of DC, Th1, Th2, Th17 and Treg in rat peripheral blood from different groups (32 days after surgery): sham surgery, renal transplant alone, renal transplant + CEPO, renal transplant + EPOR blocker + CEPO and, renal transplant + LY292004 + CEPO. The representative images of each group from 5 samples are shown and data are expressed as the mean ± SEM (^*^*p* < 0.05, p values were calculated by Student’s *t*-test).


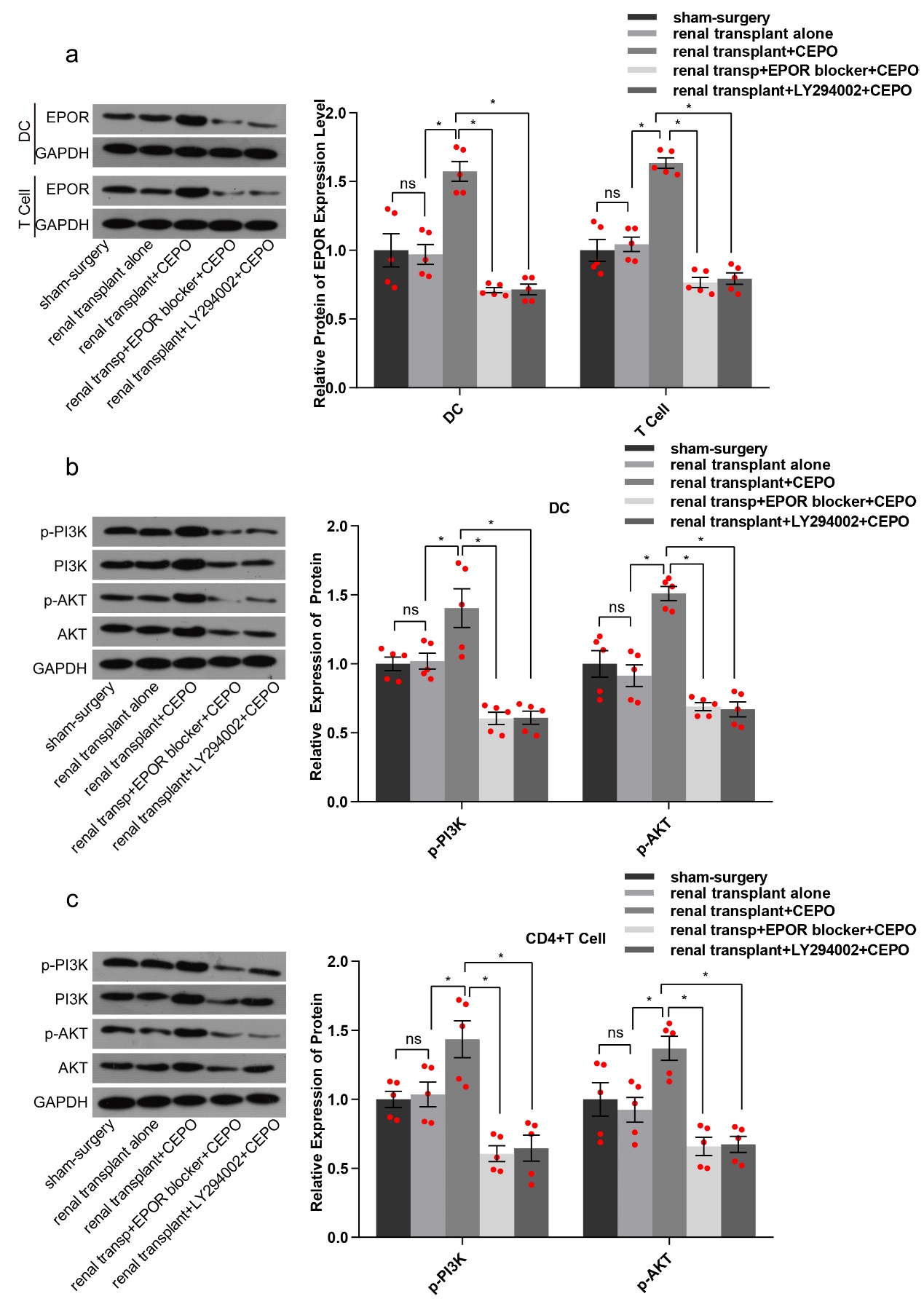


Figure S7. Influence of CEPO, EPOR blocker and PI3K/AKT inhibitor on levels of EPOR, PI3K and AKT 32 days post-transplant. Protein expression in renal transplant recipient immune cells was assessed by western blot analysis. a) Expression of EPOR in renal transplant recipient DC and CD4^+^ T cells. b) Analysis of PI3K and AKT levels in DC of renal transplant recipients. c) Analysis of PI3K and AKT levels in CD4^+^ T cells of renal transplant recipients. GAPDH was used to normalize each protein expression. The representative images of each group from 5 samples are shown and data are expressed as the mean ± SEM (^*^*p* < 0.05, p values were calculated by Student’s *t*-test).
